# Immunoproteasome Deficiency Protects in the Retina after Optic Nerve Crush

**DOI:** 10.1371/journal.pone.0126768

**Published:** 2015-05-15

**Authors:** Nathan J. Schuld, Stacy A. Hussong, Rebecca J. Kapphahn, Ute Lehmann, Heidi Roehrich, Abrar A. Rageh, Neal D. Heuss, Wendy Bratten, Dale S. Gregerson, Deborah A. Ferrington

**Affiliations:** 1 Department of Ophthalmology and Visual Neurosciences, University of Minnesota, Minneapolis, Minnesota, United States of America; 2 Graduate Program in Biochemistry, Molecular Biology and Biophysics, University of Minnesota, Minneapolis, Minnesota, United States of America; 3 Graduate Program in Microbiology, Immunology and Cancer Biology, University of Minnesota, Minneapolis, Minnesota, United States of America; 4 Histology Core for Vision Research, University of Minnesota, Minneapolis, Minnesota, United States of America; Dalhousie University, CANADA

## Abstract

The immunoproteasome is upregulated by disease, oxidative stress, and inflammatory cytokines, suggesting an expanded role for the immunoproteasome in stress signaling that goes beyond its canonical role in generating peptides for antigen presentation. The signaling pathways that are regulated by the immunoproteasome remain elusive. However, previous studies suggest a role for the immunoproteasome in the regulation of PTEN and NF-κB signaling. One well-known pathway upstream of NF-κB and downstream of PTEN is the Akt signaling pathway, which is responsible for mediating cellular survival and is modulated after optic nerve crush (ONC). This study investigated the role of retinal immunoproteasome after injury induced by ONC, focusing on the Akt cell survival pathway. Retinas or retinal pigment epithelial (RPE) cells from wild type (WT) and knockout (KO) mice lacking either one (LMP2) or two (LMP7 and MECL-1) catalytic subunits of the immunoproteasome were utilized in this study. We show that mRNA and protein levels of the immunoproteasome subunits are significantly upregulated in WT retinas following ONC. Mice lacking the immunoproteasome subunits show either a delayed or dampened apoptotic response as well as altered Akt signaling, compared to WT mice after ONC. Treatment of the RPE cells with insulin growth factor-1 (IGF-1) to stimulate Akt signaling confirmed that the immunoproteasome modulates this pathway, and most likely modulates parallel pathways as well. This study links the inducible expression of the immunoproteasome following retinal injury to Akt signaling, which is important in many disease pathways.

## Introduction

Regulation of cell signaling includes the degradation of proteins involved in the signaling cascade, and the main mediator of this process is the proteasome. The proteasome functions in many additional cell processes, such as gene expression, cytoskeletal rearrangements, and cell cycle control [1,2]. There are several distinct proteasome subtypes that regulate these processes. The standard proteasome is a multi-subunit complex that contains a 20S core particle consisting of four stacked rings of seven subunits each, with the outer rings comprised of α-subunits and the inner rings comprised of β-subunits [1,3]. The β-subunits, β1, β2, and β5, contain the active sites for proteolytic cleavage and represents the central core of catalytic activity for proteasome [3]. After translation, the newly assembling 20S core can incorporate the inducible β-subunits, LMP2 (β1i), MECL-1 (β2i), and LMP7 (β5i), to form the immunoproteasome [4]. An intermediate 20S core particle has also been identified that is comprised of both standard and immunoproteasome β-subunits [5,6]. The cellular environment drives the formation of the different proteasome core subtypes based on the need of the cell [1].

The immunoproteasome is highly expressed in immune cells and one of its main functions is to generate immunogenic peptides for major histocompatibility complex (MHC) I-restricted antigen presentation [7,8]. However, the incorporation of the inducible subunits can be promoted in non-immune tissue under conditions of cellular stress, oxidative damage, or disease, suggesting a role for immunoproteasome in functions not related to immune surveillance [1,9–13]. The immunoproteasome has also been implicated in regulating cellular signaling events, the best example being inflammatory response through NF-κB signaling [1,14,15]. In addition to NF-κB signaling, the immunoproteasome has been shown to regulate phosphatase and tensin homologue deleted on chromosome 10 (PTEN) expression, suggesting a role for the involvement of the immunoproteasome upstream of the protein kinase B (Akt) cell survival pathway [16,17].

PTEN/Akt signaling has been well studied in central nervous system (CNS) injuries, based on the role of this pathway in neuronal cell survival [18–20]. A common model to induce injury in the retina is via optic nerve crush (ONC), which initiates early and late signaling cascades eventually leading to a progressive loss of retinal ganglion cells (RGC) as well as degeneration of the optic nerve (axonal degeneration) [21]. RGC death can be instigated by diseases such as glaucoma and Huntington’s disease or by physical trauma to the retina [22–26]. Axonal degeneration in the retina is an irreversible process that can lead to blindness and has been linked to a wide variety of cell signaling pathways including PTEN/Akt.

Data from whole retinae of rats after ONC have identified that a decrease in Akt activity is parallel to a decrease in insulin-like growth factor 1 (IGF-1), suggesting the survival and death signals are meditated through the receptor tyrosine kinase (RTK) and phosphatidylinositol-4, 5-biosphosphate 3-kinase (PI3K) pathway upstream of PTEN and Akt [18]. Downstream of Akt, there are conflicting reports about the effect that nerve injury has on mammalian target of rapamycin (mTOR) signaling monitored through phosphorylation of ribosomal protein S6 (rpS6) [27,28]. More characterization of the signals that regulate this pathway after optic nerve injury is needed to develop better therapeutic options to treat this detrimental process.

The current study tests the hypothesis that the immunoproteasome modulates cell death in the retina after optic nerve injury, in part, through regulation of the PTEN/Akt signaling. The optic nerves of wild-type (WT) mice and mice deficient in one (*lmp2* ^*-/-*^) or two (*lmp7* ^*-/-*^ and *mecl1* ^-/-^) subunits of the immunoproteasome were crushed and the resulting injury was monitored through evaluation of ganglion cell layer (GCL) density and apoptosis, as well as whole retinal expression of proteins in the PTEN/Akt pathway. Furthermore, in order to assess the role of the immunoproteasome in the PTEN/Akt pathway without additional inputs, retinal pigment epithelial (RPE) cells isolated from the mice were treated with IGF-1. This current work is the first to assess the role of the immunoproteasome in a discrete injury to a limited number of specific retinal neurons via optic nerve injury, and provides new insight into the cellular stress response after ONC.

## Materials and Methods

### Mice

The mice in this study were handled in accordance with the Association for Research in Vision and Ophthalmology for the use of animals in research. Colonies of KO mice lacking one (*lmp2* ^-/-^, referred to as **L2**) or two (*lmp7* ^-/-^ and *mecl1* ^-/-^, referred to as **L7M1**) catalytic subunits of the immunoproteasome were generously donated by J. J. Monaco (University of Cincinnati). The description of gene deletions for these mice has been previously published [29–31]. Mice expressing a chimeric GFP and diphtheria toxin receptor (DTR) under the control of a transgenic CD11c promoter were also utilized for analysis of immune cells post-ONC, and were previously described [32]. Euthanasia was performed by CO_2_ inhalation followed by perfusion with PBS supplemented with 2 U/mL heparin before tissue collection. All procedures were approved by the Institutional Animal Care and Use Committee of the University of Minnesota. All mice were of the C57Bl/6 background.

### Optic Nerve Crush (ONC)

ONC was performed as previously described [21,33]. Briefly, male and female mice were deeply anesthetized with xylazine (14 mg/kg) and ketamine (60 mg/kg). A lateral canthotomy was made on one eye to access the posterior pole. The bulbar conjunctiva was cut 90° in the superior temporal region and peeled back to the posterior region of the globe. The optic nerve was exposed by gentle blunt-dissection. The nerve was clamped 1 mm from the posterior pole for 3–5 seconds using Dumont self-closing forceps (Fine Science Tools, Switzerland). Eyes were harvested from 2 to 70 days post-injury.

### Immunofluorescent Staining of Retinal Sections

Eyes were enucleated and immediately snap-frozen in Tragacanth (Sigma, St. Louis, MO). Retinas were sectioned (12 μm) through the optic nerve. For antibody staining, the tissue sections were fixed in ice cold acetone for 15 minutes, blocked for 30 minutes in 10% normal donkey serum, and then incubated in the primary antibodies overnight (Table 1). The reaction was visualized using appropriate secondary antibodies. To confirm the specificity of the primary antibody, sections were incubated in the absence of the primary antibody, using the secondary antibody alone. Antibody stained slides were cover slipped with VECTASHIELD Mounting Medium containing 4',6-diamidino-2-phenylindole (DAPI) (Vector Laboratories, Burlingame, CA).

**Table 1:** Antibodies used for Immunohistochemistry or Western Immunoblotting.

Antibody	Type	Assay	Dilution	Company
**20Sα7**	M	W	1:1000	Biomol, Plymouth Meeting, PA
**20Sβ1i (LMP2)**	P	W	1:1000	Affinity BioReagents, Golden, CO
**20Sβ5i (LMP7)**	P	W	1:1000	Biomol, Plymouth Meeting, PA
**GFAP**	M	W	1:2000	Chemicon, Temecula, CA
**GFAP**	P	I	1:100	Sigma, St. Louis, MO
**βIII-tubulin**	M	I	1:500	Chemicon, Temecula, CA
**PTEN**	M	W	1:1000	Cell Signaling, Danvers, MA
**p-Akt (T308)**	M	W	1:1000	Cell Signaling, Danvers, MA
**Akt**	M	W	1:1000	Cell Signaling, Danvers, MA
**p-S6K1 (T389)**	P	W	1:1000	Cell Signaling, Danvers, MA
**S6K1**	M	W	1:1000	Cell Signaling, Danvers, MA
**Bcl-2**	M	W	1:100	EMD Millipore, Darmstadt, Germany
**Bax**	P	W	1:1000	Cell Signaling, Danvers, MA

Monoclonal (M), polyclonal (P), Immunohistochemistry or Immunofluorescence (I), Western immunoblotting (W).

### Flow Cytometry of Immune Cells in the Retina

Mice were euthanized, perfused, and the retinas removed as described [32]. The retinas were dissociated using a solution of 0.5 μg/mL Liberase/Blendzyme3 (Roche) and 0.05% DNase in DPBS, stained with indicated antibodies, and analyzed by flow cytometry using a FACSCanto flow cytometer (BD Bioscience) [32]. Gating strategy and analysis of retinal mononuclear cells and lymphocytes has been described [33,34]. Control experiments showed that the perfusion procedure effectively removed passenger cells from the retinal vasculature so that their contribution was not significant (data not shown). For the purpose of analysis, a single sample was comprised of all cells collected from a single retina.

### TUNEL Staining (sections and whole mount)

In both retinal sections and whole mounts, detection of apoptotic nuclei was accomplished by terminal deoxynucleotidyl transferase-mediated dUTP nick-end labeling (TUNEL) using the In Situ Cell Death Detection Kit, Fluorscein (Roche, Indianapolis, IN) following the manufacturer’s instructions. Slides were cover slipped with VECTASHIELD Mounting Medium containing DAPI to visualize the nuclei.

In sections, the number of apoptotic nuclei was determined from counting the entire number of TUNEL-positive nuclei in the ganglion cell layer (GCL) of retinal sections dually-stained with TUNEL and DAPI. The averaged counts from six sections was used for each mouse and the data are reported as the number of TUNEL-positive nuclei in the outer nuclear layer per retinal section. To identify apoptotic ganglion cells in retina whole mounts, the sample was stained with βIII-tubulin followed by TUNEL.

### Retinal Morphology Measurements

The nuclei density of the GCL was measured on DAPI-stained retinal sections that were co-stained with TUNEL as described previously [33]. For each retinal section, three images were taken on either side of the optic nerve at 500 μm intervals. The length of the GCL was measured using BIOQUANT NOVA PRIME 6.90.10 (BIOQUANT Image Analysis, Nashville, TN).

### Ganglion Cell Specific Counting

Retinal whole mounts from WT and immunoproteasome KO mice were also used to quantify the number of ganglion cells in control mice and at 36 days post-ONC. Retinas were dually-stained with both βIII-tubulin and DAPI. Six separate regions were counted for each mouse (three near the optic nerve and three in the periphery). DAPI-stained nuclei were counted in the blue channel. βIII-tubulin-stained ganglion cells were counted in the green channel. The counts from the separate sections were averaged for each mouse and the percent ganglion cells were calculated by dividing βIII-tubulin positive cells by DAPI-stained total nuclei. Operators were blinded to the treatment of the mice.

### Retinal Protein Preparations

Retinas were processed as outlined [35,36], using a homogenization buffer containing 20 mM Tris (pH 7.4), 20% w/v sucrose, 2 mM MgCl_2_, 10 mM glucose, and 2% w/v CHAPS. The final supernatant containing soluble retinal proteins was stored at -80°C. Protein concentrations were determined using the BCA Assay (Pierce, Rockford, IL).

### Cell Culture

RPE cells isolated from WT or immunoproteasome KO mice (L2 and L7M1) were immortalized as previously described [37]. RPE cells were cultured in Dulbecco’s Modified Eagle Medium as described previously [15], with 5% heat-inactivated fetal bovine serum (Atlanta Biologicals). For IGF-1 treatments, cells were serum starved for 24 hours, then treated with recombinant mouse IGF-1 (100 ng/mL, R&D Systems) and harvested at times indicated in the figures. Concentration of IGF-1 utilized in the study was determined by assessing time- and concentration-dependent phosphorylated-Akt (p-Akt) activation by Western blot in WT-RPE cells from 0–3 hours and 0–300 ng/mL IGF-1, respectively (Data not shown).

### Western Blot Analysis

Western blotting was performed as described [38,39]. Membranes were incubated for 16–24 hours at 4°C with one of the primary antibodies (Table 1). Preliminary trials with the anti-MECL-1 antibody were inconsistent, so no Western blot analysis was included for this subunit. Appropriate secondary antibodies conjugated to horseradish peroxidase (Pierce, Rockford, IL) were used and immune reactions were visualized using chemiluminescence.

Immune reactions were imaged using a ChemiDoc XRS (Bio-Rad, Hercules, CA) and quantified using Quantity One (Bio-Rad, Hercules, CA) or ImageJ (NIH, Bethesda, MD). The immune reaction of a reference sample, run on each ONC blot, was used to normalize sample reactions and allowed for comparison between blots.

### Quantitative RT-PCR

Total RNA was isolated from murine retina and analysis was performed using an iQ5 Multicolor Real-time PCR I-cycler (Bio-Rad Laboratories, Hercules, CA) as described previously [15]. Reactions were performed with cDNA (6 ng/reaction, in triplicate) using 1X PCR buffer (Bioline), 3 mM MgCl_2_, 10 nM fluorescein (USB Corporation), 0.1% Triton X-100, 0.03 U/μL Immolase DNA polymerase (Bioline), 800 μM dNTP mix (Bioline), 0.025X SYBR Green (Invitrogen), and 200 nM of forward and reverse primers in a total volume of 25 μL. Normalized gene expression was determined using the iQ5 optical system software (BioRad) using acidic ribosomal phosphoprotein P0 (ARBP) expression as a reference gene for each sample. The sequence for primers used in qRT-PCR is provided in Table 2.

**Table 2:** Primers used for qRT-PCR.

GENE	Accession #	Amplicon (bP)	Primer Sequence
**LMP2 (β1i)**	**NM_013585**	**133**	Fwd 5'-CATCATGGCAGTGGAGTTG-3'
			Rev 5’-TGAGAGGGCACAGAAGATG-3’
**MECL-1 (β2i)**	**NM_013640**	**120**	Fwd 5’-AAGACCGGTTCCAGCCAAACATGA-3’
			Rev 5’-TGATCACACAGGCATCCACATTGC -3’
**LMP7 (β5i)**	**NM_010724**	**101**	Fwd 5’-GGGACAAGAAGGGACCAGGA-3’
			Rev 5’-TGCCGGTAACCACTGTCCATCA-3’
**α7**	**NP_011184**	**143**	Fwr 5’-AAACAGTAGTACAGCGATTGGG-3’
			Rev 5’-CTGCAACTGCCATTCCAACA-3’
**ARBP**	**NP_031501**	**102**	Fwd 5’-CTTTCTGGAGGGTGTCCGCAA -3’
			Rev 5’-ACGCGCTTGTACCCATTGATGA -3’

Accession number is from NCBI database. Fwr, Forward; Rev, Reverse; ARBP, acidic ribosomal phosphoprotein P0.

### Statistical Analysis

To test for statistical significance between treatment groups, data was analyzed by one-way analysis of variance (ANOVA) followed by a Dunnett’s Multiple Comparison Post-hoc test, or by a Pearson Correlation test using Graphpad PRISM version 5.00 for Windows, GraphPad Software (San Diego California, USA, www.graphpad.com) as described previously [40,41]. A Dixon’s Q-Test was performed to identify and eliminate outliers from the data. The level of significance was set at p ≤ 0.05. Data are reported as mean ± S.E. for all groups.

## Results

### ONC increases stress and immunoproteasome content in the WT retina

**Fig 1:**
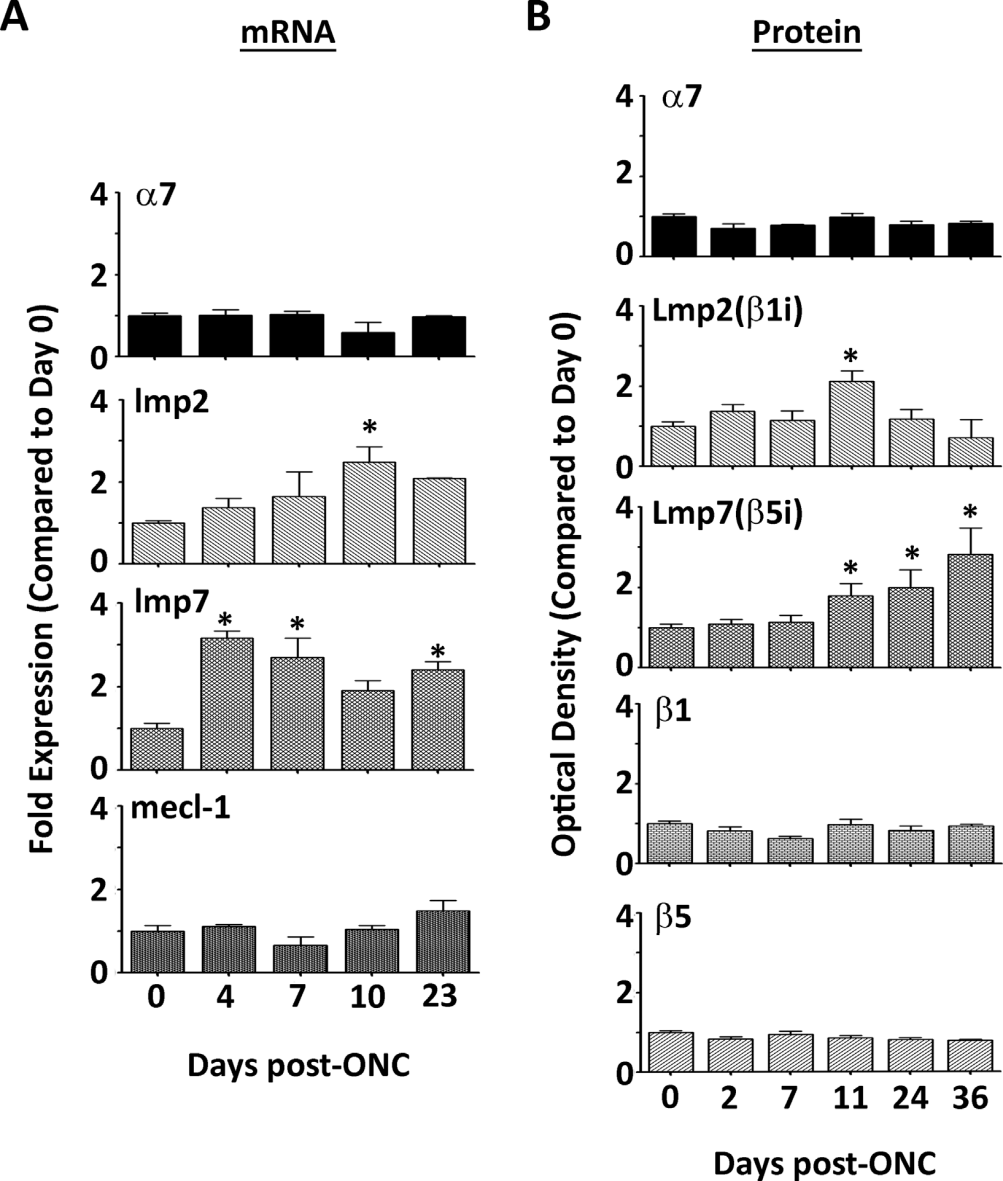
Immunoproteasome Content in the WT Retina after Optic Nerve Crush. (**A, B**) mRNA or protein was isolated from WT retinas at the indicated time points after ONC. (**A**) Results are the fold expression of the shared proteasome subunit α7 or immunoproteasome subunits lmp2 (β1i), mecl-1 (β2i), and lmp7 (β5i), and are the mean ± S.E. of 3–5** mice/group compared to control (day 0). (*, p≤0.05 by one-way ANOVA with Dunnett’s post-test compared to the ‘day 0’ values). (** All 23 day data represent 2 mice/group). (**B**) Results are the optical density of the shared proteasome subunit α7, standard proteasome subunits β1 and β5, and immunoproteasome subunits LMP2 (β1i) and LMP7 (β5i), and are the mean ± S.E. of 3–22** mice/group compared to control (day 0). (*, p≤0.05 by one-way ANOVA with Dunnett’s post-test compared to the ‘day 0’ values). (** All 36 day data represent 2 mice/group).

ONC produces a partial axotomy of the optic nerve, which results in retinal injury, quick apoptotic death, and loss of RGCs in a WT animal [21,42]. This damage is also accompanied by up-regulation of glial fibrillary acidic protein (GFAP) and ceruloplasmin, two proteins that display a well-characterized stress response [43,44]. Cytotoxic T-lymphocyte-induced injury to the retina also elicits GFAP upregulation, along with a concommitment increase in expression of the immunoproteasome, suggesting that the immunoproteasome responds to retinal injury [45,46]. To test this idea, proteasome subunit content and expression were evaluated in response to ONC. Protein and mRNA levels in the WT retina were measured using Western immunoblotting and quantitative PCR, respectively (Fig 1A and 1B).

Quantitative real-time RT-PCR was performed on WT retinae to evaluate the mRNA level in controls and at 4, 7, 10 and 24 days post-ONC (Fig 1A). No change in total message for the α7 subunit was observed and *lmp2* mRNA was upregulated at 11 days. However, *lmp7* mRNA significantly increased after only 4 days post-ONC, and remained upregulated 23 days post-ONC. The mRNA for *mecl-1*, the third immunoproteasome catalytic subunit, was unchanged. These data show that the kinetics for the mRNA expression differed between the immunoproteasome subunits after ONC and that retinal optic nerve injury leads to an increase in specific subunits of the immunoproteasome but not total proteasome.

The divergent kinetics associated with the change in mRNA content for the *lmp2* and *lmp7* subunits suggested differential protein expression of these two subunits. Protein content of the α7, β1, and β5 subunits remained stable in WT retina after ONC indicating that both total proteasome content and content of standard proteasome was unchanged following ONC (Fig 1B). In contrast, the protein levels of both LMP2 and LMP7 immunoproteasome subunits showed a significant increase in relative content following ONC in WT retina. However, similar to the mRNA expression data, the kinetics of the protein changes were different for each subunit. For LMP2, there was a ~2 fold increase in protein content at 11 days followed by a decline to control levels by 24–36 days post-ONC, which correlated to the peak seen in *lmp2* mRNA at 11 days. Contrary to the mRNA data, LMP7 in crushed eyes exhibited a time-dependent ~2–3 fold increase in protein content over control levels at 11, 24 and 36 days post-ONC. Taken together, these results suggest that ONC in the WT retina stimulates transformation of the proteasome’s catalytic core to favor incorporation of immunoproteasome subunits, although LMP2 and LMP7 expression was not coordinately regulated.

**Fig 2:**
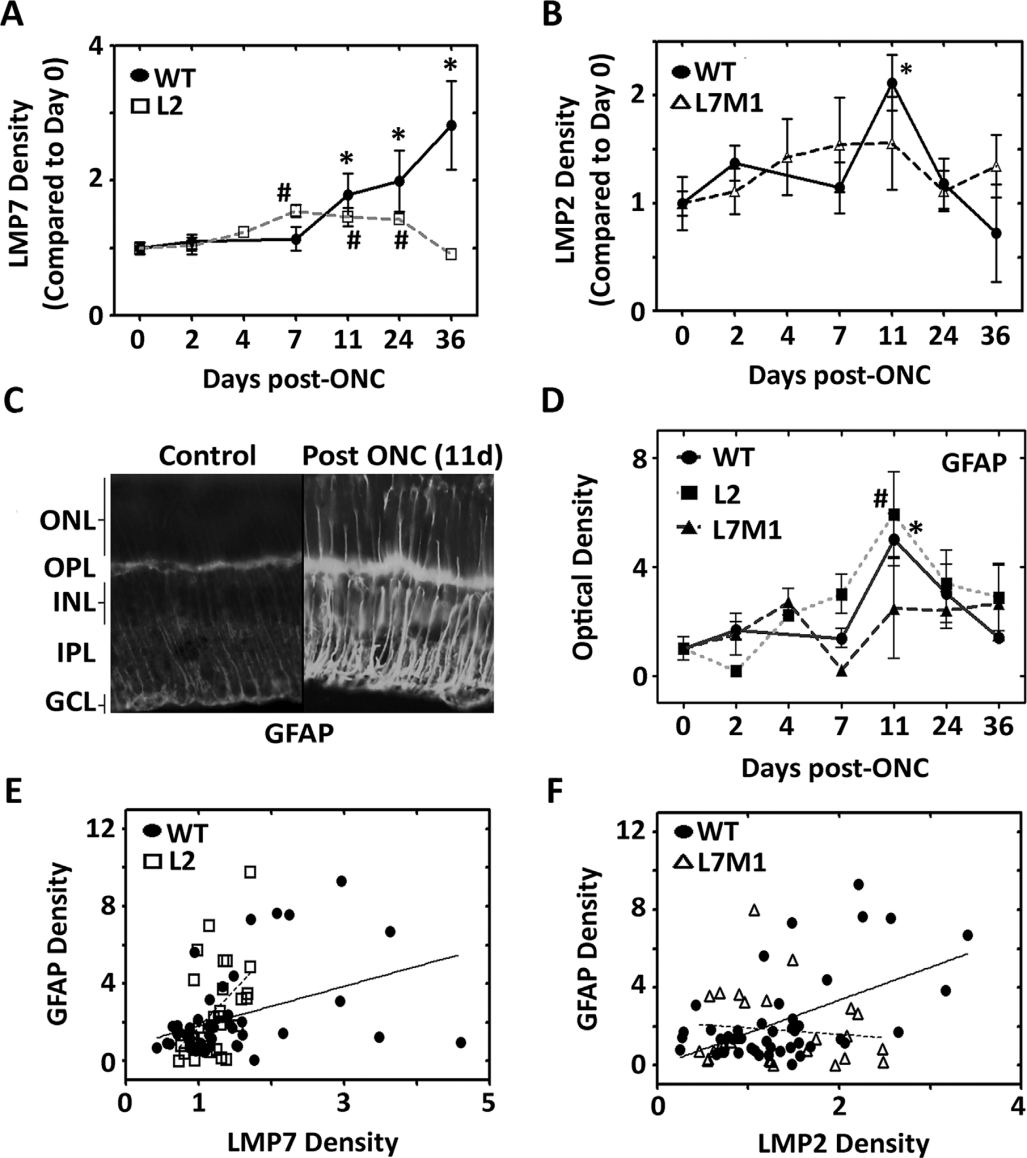
Optic Nerve Crush Increases Stress and Correlates with an Increase in Immunoproteasome Subunits in WT Retina. (**A** and **B**) Protein was isolated from WT (●), L2 (□), and L7M1 (Δ) mouse retinas at the indicated time points after ONC. Results are the optical density of immunoproteasome subunits (**A**) LMP7 (β5i) and (**B**) LMP2 (β1i), and are the mean ± S.E. of 3–22** mice/group compared to control (day 0). (*, p≤0.05 (WT), and #, p≤0.01 (L2) by one-way ANOVA with Dunnett’s post-test compared to the ‘day 0’ values). (** All 36 day WT mouse data represents 2 mice/group). (**C**) WT retina tissue sections stained with anti-GFAP in WT retina before and after optic nerve crush (11 days). Image was taken with a 40x objective. (**D**) Protein was isolated from WT (●), L2 (■), and L7M1 (▲) retinas at the indicated time points after ONC. Results are the optical density of the GFAP protein content, and are the mean ± S.E. of 3–15 mice/group compared to control (day 0) for each mouse strain. (*, p≤0.001 (WT), #, p≤0.01 (L2) by one-way ANOVA with Dunnett’s post-test compared to the ‘Day 0’ values). (**E**) GFAP and LMP7 protein content isolated from WT (●) or L2 (□) mice are shown as a correlation. (R^2^ = 0.2292; p≤0.01 (WT), R^2^ = 0.1620; p≤0.01 (L2), by Pearson Correlation). (**F**) GFAP and LMP2 protein content from WT (●) or (Δ) L7M1 mice are shown as a correlation. (R^2^ = 0.2860; p≤0.001 (WT), R^2^ = 0.0126, p = 0.57 (L7M1), by Pearson Correlation).

Immunoproteasome subunits were also monitored in KO retinas. Similar to the response in WT retina, L2 mice also showed a significant increase in LMP7 content from 7–24 day post-ONC, whereas L7M1 mice did not show a significant increase in LMP2 content post-ONC (Fig 2A and 2B). The standard proteasome subunits, β1 and β5, and the shared proteasome subunit α7 did not change post-ONC in the immunoproteasome KO mice, indicating no change in total proteasome content after injury (S1 Fig). These data provide evidence that the ablation of both LMP7 and MECL-1 prevents a stress-induced increase of the LMP2 subunit, but the ablation of LMP2 alone does not prevent a stress induced increase in the LMP7 subunit.

To verify the retinal stress caused by ONC, the content of GFAP was evaluated from WT, L2, and L7M1 mouse retinae before ONC and up to 36 days after ONC. GFAP was significantly upregulated in WT mice 11 days after ONC as shown on retinal sections by immunohistochemistry, and by Western blotting of protein from whole retina (Fig 2C and 2D). GFAP was also significantly upregulated in the retina of L2 mice, but not in the retina of L7M1 mice suggesting that the absence of specific immunoproteasome subunits alters the retinal response to stress.

The correlation between GFAP and immunoproteasome subunit content post-ONC in individual mice was next assessed in the WT, L2, and L7M1 retinas (Fig 2E and 2F). LMP7 content significantly correlated with GFAP expression in both the WT (R^2^ = 0.1620, p<0.01) and L2 (R^2^ = 0.2295, p<0.01) retina (Fig 2E). The content of LMP2 significantly correlated with GFAP in the WT retina (R^2^ = 0.2860, p<0.001), but not in the L7M1 retina (R^2^ = 0.0126, p = 0.57) (Fig 2F). Furthermore, the correlative increase of the immunoproteasome subunits concomitantly with GFAP supports a role for the immunoproteasome similar to GFAP, as an indicator of retinal stress.

**Fig 3:**
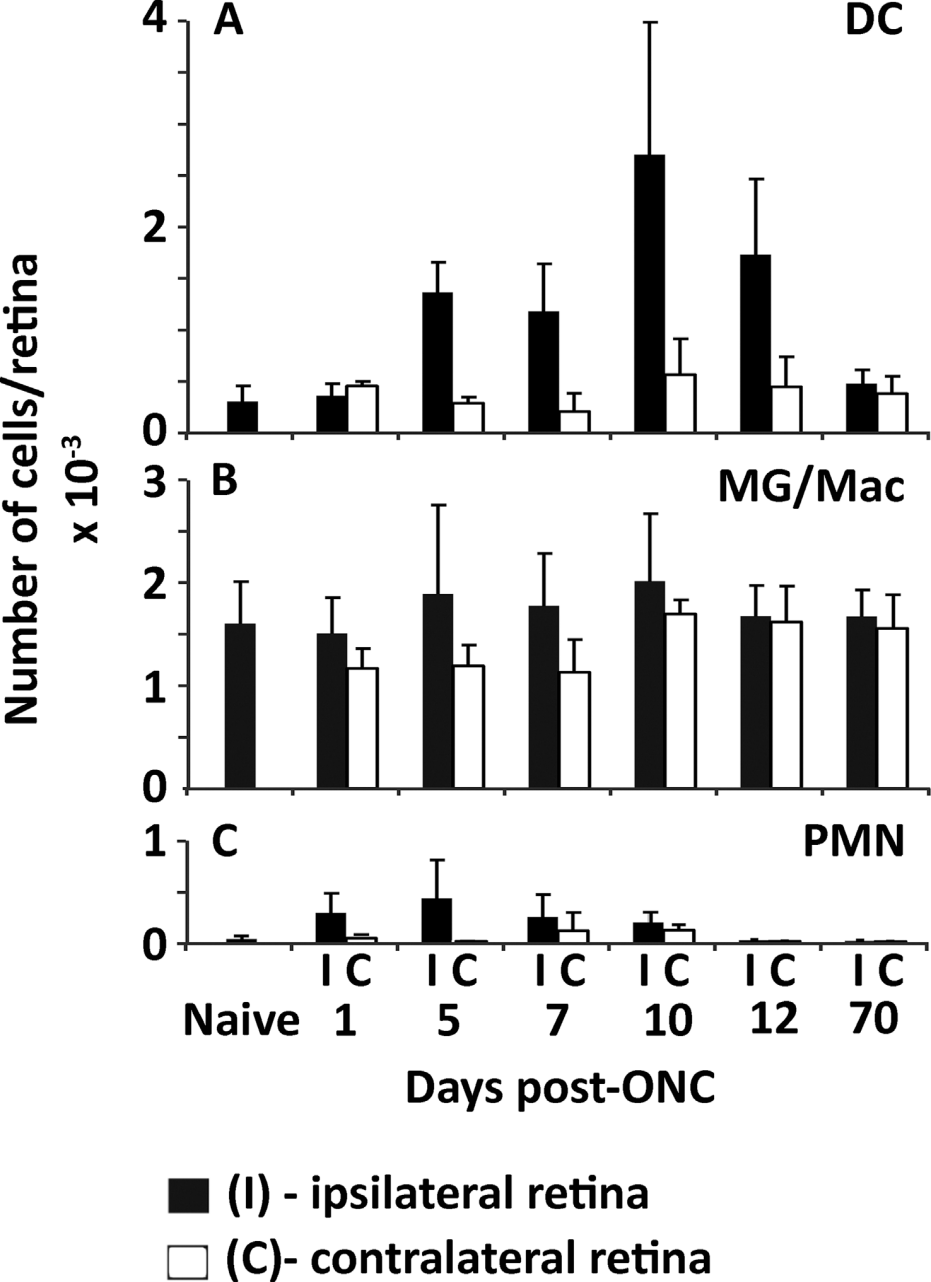
Mononuclear cell response in mouse retina post-ONC. Recruitment of CD45+ cells to retina in CD11c-DTR/GFP mice on the B6 background. Retinas were harvested from naive mice, and from mice at intervals ranging from 1 day to 10 wks post-ONC. (**A**) Dendritic cells (DC) were identified as CD45+CD11b+Ly6G-GFP+. (**B**) Microglia/macrophages (MG/Mac) were identified CD45+CD11b+Ly6G-GFP-. (**C**) Neutrophils (PMN) were CD45+CD11b+Ly6G+GFP-. Other immune cells were present in very small numbers, and not significantly changed by the ONC. Closed bars—ipsilateral (ONC-treated) retinas; open bars—contralateral control retinas. Results are the mean ± S.D. of 4–5 mice/group.

Previous work has reported a time dependent influx of immune cells into the retina following ONC [47]. Since the immunoproteasome is highly expressed in immune cells [1] we assessed the populations of dendritic cells (DC), microglia/macrophages, and polymorphonuclear leukocytes (PMN) to determine their relative contribution to our Western blotting results (Fig 3). The lack of antibodies for CD11c, a marker of DC, required that we utilize a genetic model expressing GFP under control of the CD11c promoter to track CD11c positive cells in the retina. The optic nerves from mice expressing GFP on the CD11c promoter were crushed and the ipsilateral (I) and contralateral (C) retinal cells were stained for macrophages and PMN cells and sorted by flow cytometry. Dendritic cells in the crushed retina showed an increase from ~250 cells in naïve retina to ~2500 cells by 10 days post-ONC with a return to baseline after 10 weeks (Fig 3A). Both microglia/macrophages and PMNs did not change significantly over the post-ONC period for ipsilateral and contralateral retina (Fig 3B and 3C). Immunoproteasome subunit content in the contralateral retinae from our WT and immunoproteasome KO mice did not change significantly over time post-ONC (data not shown).

Overall, two factors suggest that the immune cells contribute little to the total immunoproteasome content of the retina. 1) Immune cells express the immunoproteasome constitutively[48,49], and 2) the contribution of the immune cells for immunoproteasome content measured in the whole retina post-ONC can be considered insignificant based on the total cell amounts present. For example, previous literature estimates that in a mouse retina there are over 8 x 10^6^ cells total [50], and our data indicates that at most there are around 5000 total immune cells accounting for less than 0.1% of the population. Based on these observations, our data supports the hypothesis that the immunoproteasome content increase seen post-ONC was occurring in the non-immune cells of the retina.

### Immunoproteasome deficiency results in altered cell death in the GCL after ONC

**Fig 4:**
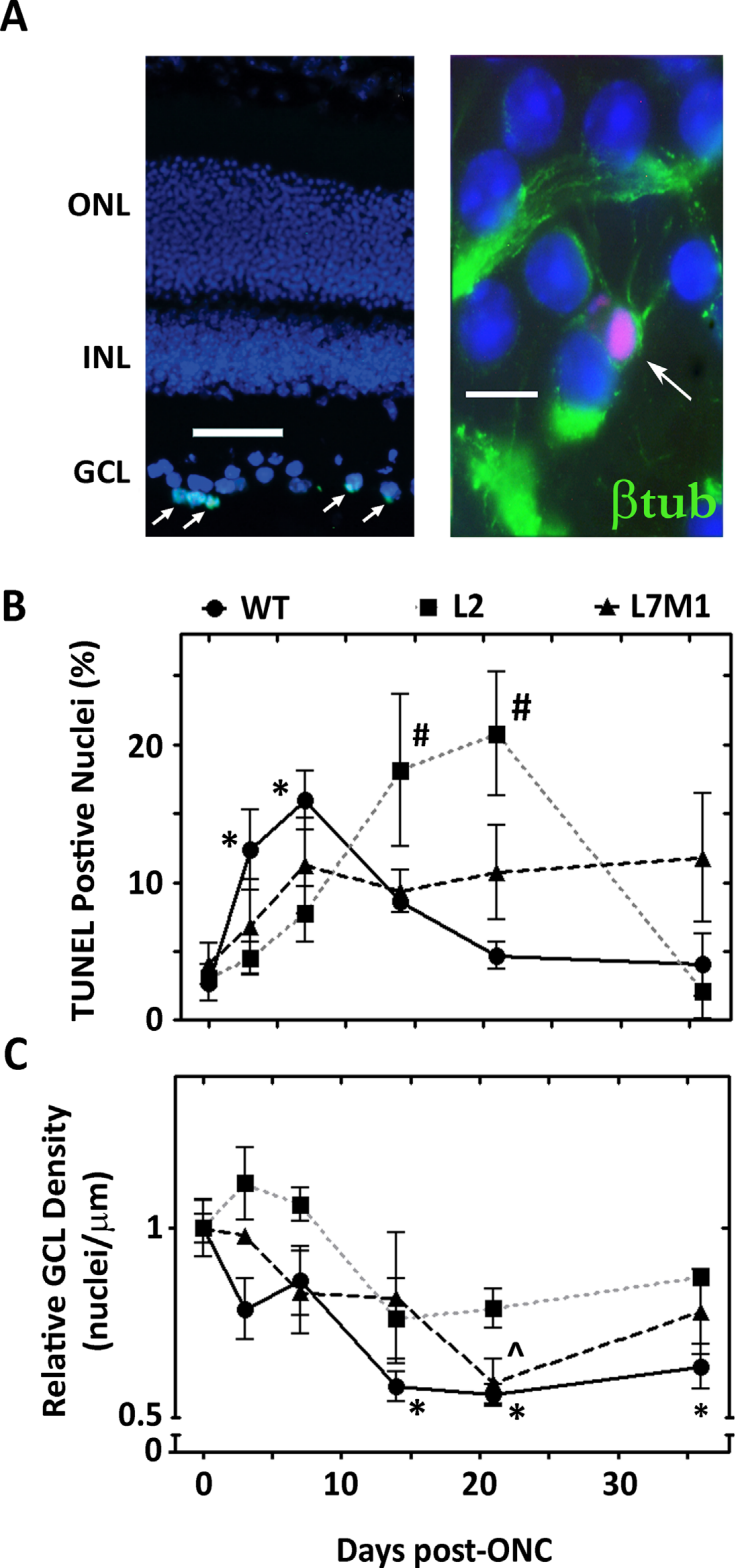
Immunoproteasome Deficiency Results in Altered Ganglion Cell Death after ONC. (**A, *left***) Representative retinal section 11 days following ONC stained with DAPI (blue) and TUNEL (green). GCL, ganglion cell layer; INL, inner nuclear layer; ONL, outer nuclear layer. Image was taken with a 20x objective and the scale bar indicates 50 μm. (**A, *right***) Representative retinal whole mount stained with anti-βIII-tubulin (green), TUNEL (red, arrow), and DAPI (blue). Image was taken with 100x objective and the scale bar indicates 10 μm. (7 days following ONC) (**B**) Summary of the percent TUNEL-positive cells in the GCL following ONC from WT (●), L2 (■), and L7M1 (▲) mice. Results are the mean ± S.E. of 3–9 mice/group compared to control (day 0). (*, p≤0.01 (WT) and #, p≤0.05 (L2) by one-way ANOVA with Dunnett’s post-test compared to the ‘day 0’ values). (**C**) Summary of GCL nuclei density (nuclei/μM) following ONC from WT (●), L2 (■), and L7M1 (▲) mice. Results are the mean ± S.E. of 3–9 mice/group compared to control (day 0). (*, p≤0.01 (WT) and ^, p≤0.05 (L7M1) by one-way ANOVA with Dunnett’s post-test compared to the ‘day 0’ values).

To monitor the time course of ONC-induced apoptosis and subsequent loss of cells in the GCL, DNA fragmentation was assayed using TUNEL staining and nuclei were counted in the ganglion cell layer of DAPI-stained retinal sections from controls and at 3 to 36 days post crush (Fig 4B). Representative DAPI- and TUNEL-stained retinal sections (11 days post-ONC) shows apoptotic cells in GCL (Fig 3A (*left*), and retinal whole mount (7-days post-ONC) stained with DAPI, TUNEL (red), and βIII-tubulin (green) indicate that ganglion cells in this layer are apoptotic (Fig 3A (*right*). Consistent with previous GCL death data in WT rats and rabbits [18,23], a peak in GCL TUNEL staining at 3 and 7 days post-crush followed by a gradual decrease in the number of TUNEL-positive nuclei by day 21 post-ONC was observed (Fig 4B). Mice lacking the LMP2 subunit displayed a delayed apoptotic response post-ONC compared to WT mice, with a peak at 14 and 21 days and a return to baseline by 36 days (Fig 4B). The overall apoptotic response was dampened in L7M1 mice, resulting in no significant increase from baseline, although there was a consistent apoptotic signal that persisted throughout the 36 days post ONC (Fig 4B).

Counts of nuclei present in the WT GCL showed a rapid decrease in nuclei during the first week post-crush, with an approximate 44% loss in nuclei by 14 days which temporally correlated with the TUNEL data (Fig 4C). Maximum cell loss had likely occurred by 21 days based on (i) the consistent number of nuclei observed in our samples at 14 and 36 days and (ii) previous reports of stable numbers of nuclei in the GCL from 3 to 6 weeks post ONC [21]. Mice lacking the LMP2 subunit showed no significant change in GCL density. However, there was a trend towards a decrease in density 14–36 days post-ONC (Fig 4C). For L7M1 mice, a gradual decrease in GCL cell density that was significantly less at day 21 post-ONC was observed (Fig 4C). The delayed/dampened response of apoptosis and loss of nuclei in the GCL in retinas lacking the immunoproteasome subunits suggest that the immunoproteasome deficient mice have a more robust cell survival response compared to WT mice after optic nerve injury. The altered kinetics could also be due to compromised clearance of apoptotic dead cells by macrophages in immunoproteasome deficient mice [51].

One limitation of the method used in this study is analyzing all of the cells in the GCL. Previous work demonstrated that ~59% of displaced amacrine cells also reside in this layer and have been shown to be coupled to ganglion cells for signaling [50]. To confirm the specific loss of ganglion cells, we counted βIII-tubulin positive nuclei in control mice and at 36 days post-ONC in retinal whole mounts where only WT nuclei were significantly less from the WT control (Fig 4C). On average in control WT, L2, and L7M1 mice, ~30% of the total nuclei in the GCL were ganglion cells (S2 Fig). At 36 days post-ONC there was significantly fewer ganglion cells in WT, L2, and L7M1 mouse retinae (~60%, 45%, and 59% decrease, respectively) compared with the uncrushed controls (S2 Fig). The significant loss of ganglion cells in the GCL (S2 Fig), but no significant loss of overall GCL nuclei density (Fig 4C) in L2 and L7M1 mice 36 days post-ONC suggests that immunoproteasome deficiency could affect cell signaling between ganglion cells and their coupled cells in the retina [52].

### Immunoproteasome deficiency alters content of stress proteins after ONC

**Fig 5:**
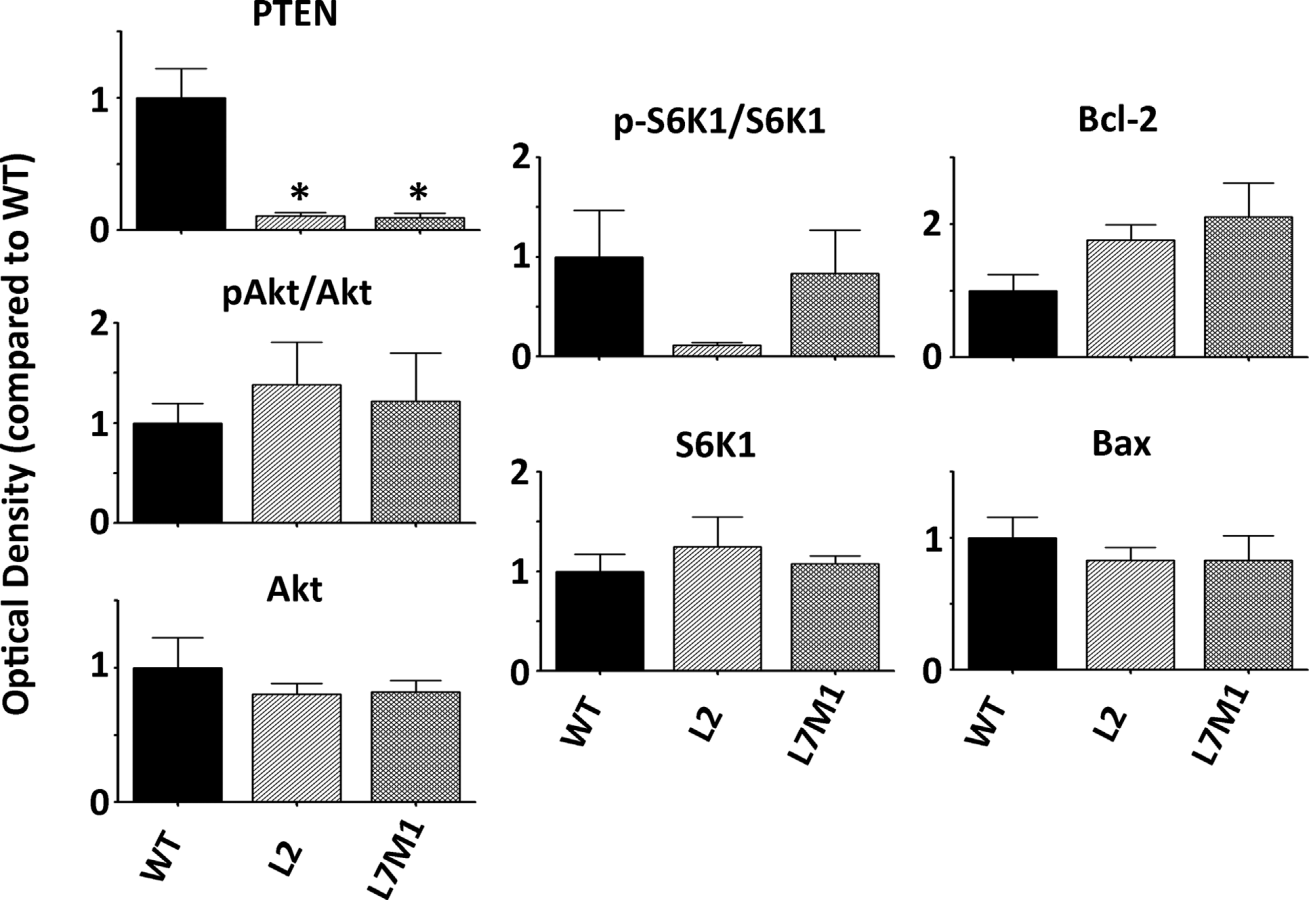
Protein Content in WT, L2, and L7M1 Murine Retinas before Injury. The optical density of protein content from uncrushed WT, L2, and L7M1 mouse retinas normalized to WT mice. Protein content is shown for PTEN, p-Akt/Akt, Akt, p-S6K1, S6K1, Bcl-2, and Bax. Results represent the mean ± S.E. of 4–9 mice/group. (*, p≤0.001 by one-way ANOVA with Dunnett’s post-test compared to WT mouse retina).

**Fig 6:**
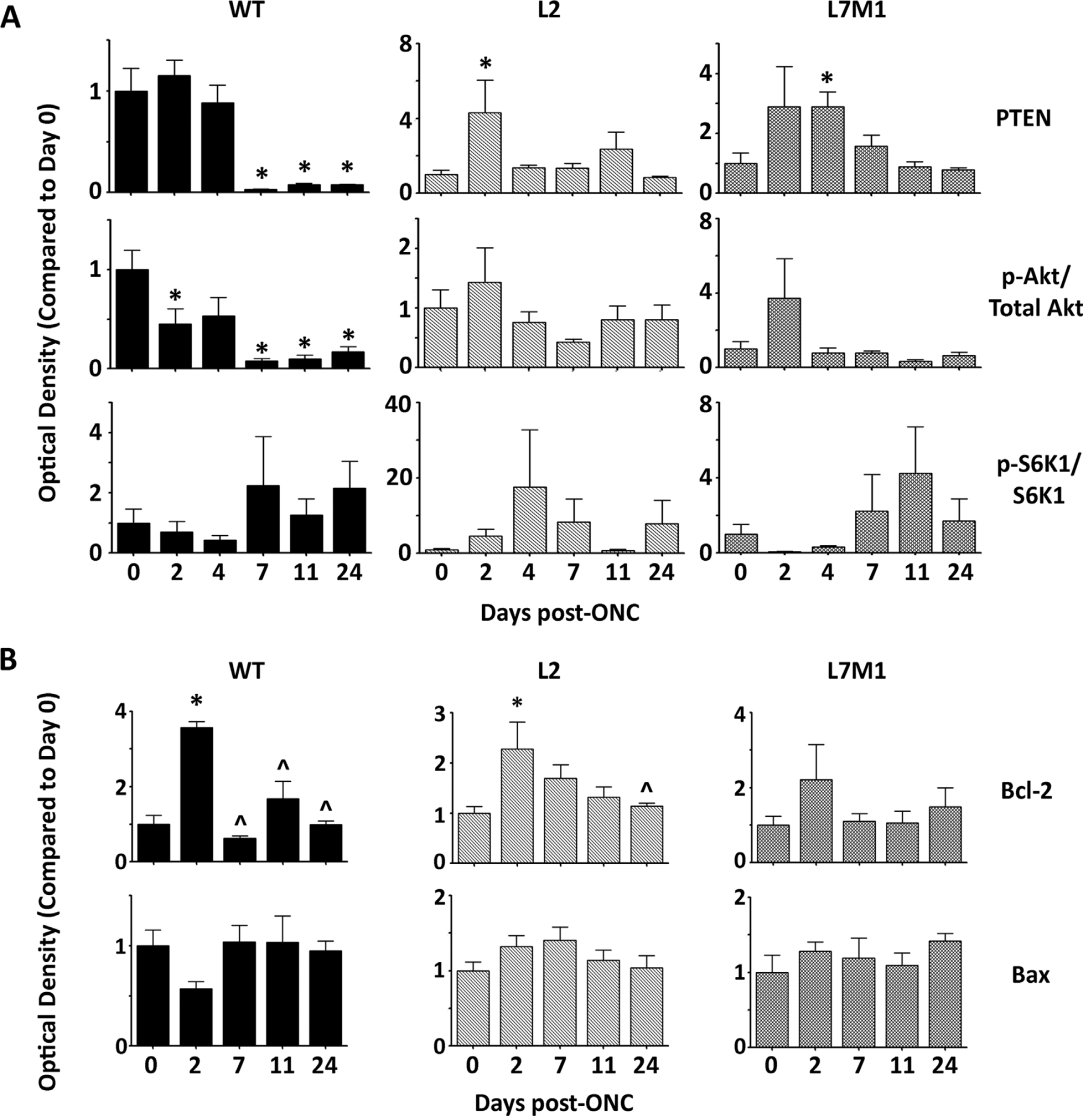
Immunoproteasome Deficiency Alters Content of Stress Proteins after ONC. (**A** and **B**) Protein was isolated from WT, L2, or L7M1 retinas at the indicated time points after ONC. Results are the optical density of PTEN, p-Akt/Akt, and p-S6K1/S6K1 (**A**), or Bcl-2 and Bax (**B**) protein content. Data is shown relative to each mouse strains’ control (day 0) and represent the mean ± S.E. of 3–9 mice/group. (*, p≤0.05 by one-way ANOVA with Dunnett’s post-test compared to the ‘day 0’ values or (^, p≤0.05 by one-way ANOVA with Dunnett’s post-test compared to the ‘day 2’ values).

The survival signaling that occurs in the whole retina was next assessed to understand the role of the immunoproteasome after ONC. An important pathway that regulates cell survival involves the PTEN/Akt signaling axis found downstream of the IGF receptor (IGFR) and PI3K pathway. Previous studies have demonstrated the involvement of survival proteins such as Akt, PTEN, and Bcl-2 family members in cellular response after optic nerve crush. Therefore, we tested the hypothesis that the immunoproteasome modulates signaling of these proteins in the retina after optic nerve injury. Protein content of PTEN, p-Akt (T308), total Akt, total p70 S6 Kinase (S6K1) (a target of mTOR activity), p-p70 S6 Kinase (T389) (p-S6K1), Bcl-2, and Bax were measured by Western immunoblotting from WT, L2, and L7M1 mice prior to and following optic nerve injury (Figs 5 and 6).

Basal levels of PTEN were significantly lower in L2 and L7M1 mice when compared to WT mice prior to injury (Fig 5). All of the other proteins that were assessed did not show a significant difference in L2 or L7M1 mice compared with WT mice. The absence of any major difference in the basal content of cell survival proteins between WT and immunoproteasome deficient mice supports our hypothesis that the immunoproteasome is involved in the stress response, but has minimal impact in the non-injured retina. We observed that optic nerve injury stimulated an increase in the expression of the immunoproteasome and we hypothesize that this increase results in modulation of signaling pathways after injury.

Optic nerve injury in WT murine retina resulted in a significant decrease in PTEN and p-Akt (T308) protein expression, but no significant change in p-S6K1 expression (Fig 6A). Anti-apoptotic Bcl-2 protein expression was significantly upregulated in the WT retina just 2 days post-ONC, followed by a rapid return to baseline from 7–24 days post-ONC (Fig 6B). There was no significance difference in pro-apoptotic protein Bax in the WT retina (Fig 6B).

Immunoproteasome KO mice showed a different PTEN expression profile post-ONC in comparison to WT mice. The results demonstrate a significant increase in PTEN expression 2 and 4 days post-ONC, in L2 and L7M1 mice respectively, followed by a return to baseline levels (Fig 6A). P-Akt/Akt levels were also different in L2 and L7M1 mice when compared to WT mice post-ONC, showing no significant protein expression change over time (Fig 6A). Downstream of p-Akt, there was no significant change in Bax or p-S6K1 kinase protein levels in L2 or L7M1 mice post-ONC similar to the WT mice (Fig 6A and 6B). Bcl-2 protein levels in the L2 retina increased similarly to the WT retina 2 days post-ONC. However, pro-survival Bcl-2 levels remained elevated until 24 days post-ONC, correlating with the delay in apoptosis seen in the ganglion cells of L2 retina (Figs 4B and 6B). In contrast, there was no significant change in Bcl-2 protein levels in L7M1 retina post-ONC, which could explain why there was a dampened apoptotic response in L7M1 mice (Figs 4B and 6B).

Taken together, these data suggest that the immunoproteasome plays a role in modulating select proteins of the PTEN/Akt signaling pathway after ONC. Previous reports demonstrate that a decrease in the growth factor IGF-1 after ONC mediates the signaling through the PTEN/Akt pathway [18]. Therefore these data led to a secondary hypothesis that the immunoproteasome modulates IGF/Akt signaling and this could contribute to the difference in cell death between the WT and immunoproteasome KO mice.

### Immunoproteasome deficiency alters temporal IGF-1 Signaling

**Fig 7:**
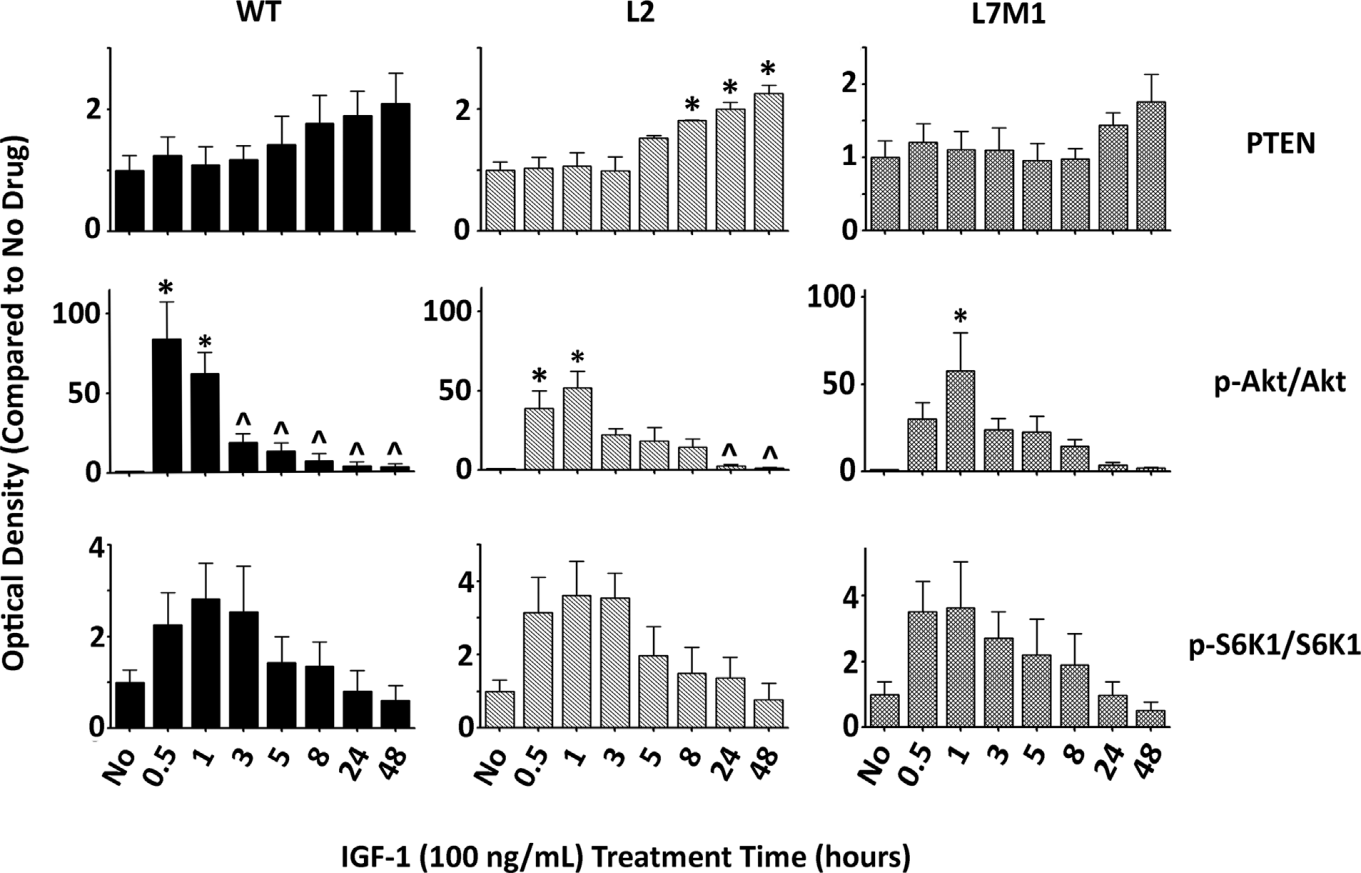
Immunoproteasome Modulates RPE Cell Signaling after IGF-1 Stimulation. RPE cells isolated from WT, L2, and L7M1 mice were serum starved for 24h and treated with IGF (100ng/mL) for the indicated time points. Results are the optical density of PTEN, p-Akt/Akt, and p-S6K1/S6K1 protein content. Data are shown as a proportion of each mouse strains’ control (day 0) and represent the mean ± S.E. of 3–6 repeats. (*, p≤0.05 by one-way ANOVA with Dunnett’s post-test compared to the ‘No Drug’ values; ^, p≤0.05 by one-way ANOVA with Dunnett’s post-test compared to the ‘30 min’ values.

To isolate IGF-1 signaling and test the hypothesis that this pathway is modulated by the immunoproteasome, RPE cells from WT, L2, and L7M1 mice were used. To eliminate excess growth factors, the cells were serum-starved overnight followed by addition of recombinant mouse IGF-1, and sampled at time points from 30 minutes to 48 hours. The content of proteins involved in the PTEN/Akt signaling pathway were then characterized by Western blot (Fig 7) (*Representative western blots are shown in* S3 Fig). PTEN expression during the treatment of the RPE cells with IGF-1 revealed no significant changes over 48h in WT and L7M1 cells. However, a significant increase in PTEN expression was observed from 8h-48h after IGF-1 addition to L2-RPE cells (Fig 7).

Addition of IGF-1 to WT-RPE cells resulted in a significant increase in the ratio of p-Akt/Akt (30m, 1hr), followed by a significant rapid decline in expression after 3-48hrs. L2-RPE cells demonstrated a similar activation of p-Akt at 30m and 1hr, however the decrease in p-Akt expression was delayed and showed a decline only after 24hrs (Fig 7). In contrast, the activation of p-Akt was delayed in L7M1-RPE cells and no significant decrease in protein expression was observed over 48hrs. The ratio of p-S6K1/S6K1 kinase expression revealed similar kinetics in an increase followed by decrease of expression for WT-, L2-, and L7M1-RPE cells indicating that the temporal differences observed upstream were not translated downstream through mTOR signaling, similar to the lack of effect on mTOR signaling after ONC (Fig 7).

Taken together these data suggest that the immunoproteasome modulates Akt signaling upstream, however the temporal modulation does not seem to translate downstream through mTOR signaling. These results suggest that the immunoproteasome does not solely affect the Akt pathway, but affects other pathways after optic nerve injury that contribute to cell death. Furthermore, the accumulation of signals from multiple pathways likely contributes to the difference in regulation of cell death seen in mice deficient in the immunoproteasome.

## Discussion

This study investigated the role of the immunoproteasome in optic nerve injury, focusing on (1) the survival of cells in the GCL, (2) protein changes seen in the whole retina and (3) signaling affected by the immunoproteasome during axonal degeneration in the whole retina. (4) Additionally, these *in vivo* studies were supplemented by isolating a specific survival pathway (Akt/PTEN) in RPE cells isolated from our WT and KO mice. Our central hypothesis is that the immunoproteasome assists in the apoptotic and cell survival signaling cascades after injury to the optic nerve. In the current study, we used an optic nerve crush injury model that has been utilized extensively in research in order to mimic axonal degeneration seen in disease and trauma [24,53–55]. The characterization of signaling events after ONC in the retina, while numerous, still need more exploration in order to develop effective therapies to combat this form of injury. Therefore, we focused on signaling through the PTEN/Akt signaling axis following ONC both in WT mice and in immunoproteasome KO mice to provide further mechanistic insight. We show for the first time that the immunoproteasome participates in these signaling pathways after optic nerve injury, and ablation of the immunoproteasome has protective effects after this type of injury.

Consistent with previous reports, we show that two immunoproteasome subunits, LMP2 and LMP7, are upregulated after injury most likely in the non-immune cells of the retina [12,45,56]. There was no change in total proteasome (α7) content or expression, nor was there a change in standard proteasome content or expression (β1 and β5) indicating that optic nerve injury only increases the immunoproteasome subunits of the proteasome. These observations correlate with proposed broad functions of the immunoproteasome after exacerbation of the cell to include: regulation of cellular homeostasis [39,57], protection from oxidative damage [58,59], and inflammation response [15,57], all processes modulated by ONC [54,60,61].

With ONC injury, the different kinetics of expression exhibited by LMP2 and LMP7 suggests differential regulation of these subunits that results in increased heterogeneity of 20S cores. The existence of core particles containing a mixture of both standard and immunoproteasome subunits are possible because incorporation of LMP2 and LMP7 into the core particle is not mutually dependent [62]. Other examples of differential regulation of subunit expression include the significant, exclusive, upregulation of LMP2 in cells following oxidative stress [59,63], and in the inclusion bodies of myositis muscle [64]. In an animal model of long-lasting renal ischemia and subsequent renal atrophy, only the LMP7 subunit was upregulated [65]. It is therefore possible in our KO mice that the immunoproteasome subunit(s) not silenced form a hybrid proteasome. The production of a hybrid proteasome could explain why we see altered functions or signaling between the two different KO mouse models. This finding supports emerging evidence that each subunit of the immunoproteasome can affect not only similar, but dissimilar pathways that are entirely dependent on the cell, organ, tissues, or injury [66]. More extensive studies in this area are needed, however, to fully comprehend the consequence of generating hybrid proteasomes and the resulting effects on cell function.

A critical question in proteasome research is whether the immunoproteasome is protective or detrimental to the course of disease pathogenesis or response to injury? There are numerous reports supporting a protective role for the immunoproteasome in the cell through clearance of oxidatively damaged proteins [59], preservation of cellular homeostasis after inflammation [57], assistance with adipocyte maturation [67], protection from progression of diabetes [68], and regulation of tumor development [69]. Alternatively, there is a more limited repertoire of reports demonstrating a detrimental role for the immunoproteasome. These studies show that inhibition of the immunoproteasome protects from inflammation in experimentally produced colitis [70], reduces inflammatory symptoms of experimental arthritis [71], and prevents prostrate tumor growth in mice [72]. Our results demonstrate that deficiency of LMP2 alone, or both LMP7 and MECL-1 in mice, delays or protects against ONC-induced apoptosis seen in the GCL (Fig 4).

We observed differences in GCL loss between WT and immunoproteasome KO mouse retinae demonstrating no significant change in GCL cell loss in the L2 mice and minimal cell loss in the L7M1 mice (Fig 4C). We isolated specific ganglion cell loss in the GCL, and our data demonstrates that there is a significant decrease of ganglion cells 36-days post-ONC in both WT and immunoproteasome KO mice. These data indicate that immunoproteasome deficiency does not ultimately prevent ganglion cell death but rather affects the temporal onset of apoptosis (S2 Fig). One explanation for the difference between ganglion cell loss and total nuclei content of the GCL, could be due to the population of cells in the GCL comprising of both ganglion cells and displaced amacrine cells [50]. The ganglion cells and amacrine cells have been shown to coordinate death signaling after injury via gap junctions [52]. It is intriguing to hypothesize that retina deficient in specific immunoproteasome subunits impair the ability of ganglion cells to send the death signal to the coupled amacrine cells after optic nerve injury, which could lead to a better overall cell survival in the retina. However, more studies are needed to tease out the differences in singling between these two cell types in the GCL.

After ONC, damaged or dying ganglion cells are removed either through resident microglia or recruited macrophages [51]. Part of the process for clearance of dying cells by phagocytosis involves signaling via the NF-κB pathway, among others [73]. The immunoproteasome has been linked to modulation of the NF-κB pathway through several studies [15,74]. Consistent with NF-κB regulation of phagocytic clearance by immune cells is the observation that recruitment of macrophages and dendritic cells is substantially reduced in mice deficient in NF-κB upstream modulators TRIF and MyD88 [33]. Therefore, previous literature and data in the current study suggest that the immunoproteasome participates in either promoting cellular death signals or suppressing cellular survival signals after injury, and supports a role for the immunoproteasome in axonal degeneration and clearance by immune cells. More studies are needed however, to differentiate the effect that the immunoproteasome has on these cellular processes.

Axonal regeneration versus axonal degradation as seen in amphibians versus mammals respectively, is studied to determine the signals that lead to these very different outcomes [18,19]. Although there have been numerous studies on optic nerve injury and potential treatments to prevent axonal degeneration or promote axonal regeneration, there is a surprising lack of information regarding long term protein expression profiles in the whole retina after injury. This study sought to paint a more complete picture of the environment of the retina after an induced injury. Although most of our ONC data from the WT murine retina agreed with previous reports, there were some differences.

The kinetics of p-Akt and Bcl-2 protein expression after injury in the WT retina correlate to a previous report on rat retinas after optic nerve crush, however our Bax protein expression post-ONC were not consistent with findings from this study (Fig 6) [18]. Very few studies focused on PTEN protein expression post-ONC, however one report identified a significant decrease in PTEN mRNA levels, but not protein levels, 3 days post-ONC [75]. We saw a striking decrease in PTEN expression 7–24 days post crush that could coincide with the decrease in PTEN mRNA levels discussed above (Fig 6). The phosphatase function of PTEN removes a phosphate from phosphatidylinositol (3,4,5)-triphosphate (PIP_3_), thereby removing a docking/activation site for 3-phosphoinositide dependent protein kinase-1 (PDK1), the kinase that phosphorylates and assists in the activation of Akt [76]. Following this signaling paradigm, we expected to see the decrease in PTEN protein levels shown in this manuscript to result in an increase in p-Akt levels. However, our results show a concomitant decrease in p-Akt along with PTEN in WT retina post-ONC (Fig 6). One possible explanation could be that the cell and surrounding microenvironment had already been committed to cell death prior to 7 days post-crush, and the extracellular signals did not support signaling through this pathway regardless of PTEN expression. This argument is bolstered by the TUNEL data showing that apoptosis peaks at 3–7 days in WT mice, and a previous study that shows IGF-1 is quickly downregulated in the GCL of rats after ONC [18].

As discussed earlier, the immunoproteasome, specifically the subunit LMP2, has been linked to the regulation of PTEN in cardiac muscle [16,17]. Our data from the WT murine retina supports this hypothesis, demonstrating an increase in the expression and content of the immunoproteasome subunits from 4–36 days that corresponded with a decrease in PTEN protein expression from 7–24 days (Figs 1 and 6). Furthermore, in L2 and L7M1 mice, PTEN content increased after ONC suggesting that the absence of specific immunoproteasome subunits in the retina either promotes transcription of PTEN or increases the half-life of this protein (Fig 6). We also observed a significant increase in PTEN content in L2-RPE cells over time (Fig 7). Contrary to these data, PTEN content was significantly lower in L2 and L7M1 mouse retinas compared to the WT mice prior to crush injury. However, the expression of the immunoproteasome in WT mice was also not induced at this timepoint.

More recently, PTEN has been targeted as a therapeutic option in axon regeneration with reports demonstrating that inhibition or deletion of PTEN promotes regeneration after optic nerve injury [27,77]. It is intriguing to speculate that PTEN expression prior to injury is what ‘primes’ the cell for its fate after injury. Data from the L2 and L7M1 mice supports this idea. The basal PTEN content is initially lower and these mice have better outcome in that cell death is delayed or dampened after injury. This delay in cell death increases the therapeutic window and may allow for the opportunity for further therapeutic interventions that will ultimately inhibit RGC death and prevent axonal degeneration. Further studies are needed to develop the relationship between PTEN and the immunoproteasome in order to fully elucidate these mechanisms.

Axonal regeneration through inhibition of PTEN has been linked to mTOR activity, although the signaling events after injury between PTEN and mTOR have yet to be elucidated [27,78]. Previous neuronal injury studies focused on monitoring mTOR activity through the phosphorylation of rpS6 that is downstream of S6K1, with one study showing an increase and another showing a decrease in p-rpS6 levels one day after injury [27,28]. Our study focused on phosphorylation of S6K1 as a readout for mTOR activity, and found no significant changes following ONC in WT, L2, and L7M1 mice. After activation of the IGF-1 pathway in RPE cells from these mice, we saw a rapid phosphorylation of S6K1 followed by a gradual decrease to baseline in all three cell lines. These data indicate that the temporal change in p-Akt we observed in the immunoproteasome-deficient mice was not relayed through mTOR signaling downstream of this pathway. These data suggest that the immunoproteasome is likely functioning through other pathways in addition to PTEN/Akt.

Although stimulating RPE cells with IGF-1 (Fig 7) does not mimic optic nerve injury, our ONC data along with previous literature [18] led to a secondary hypothesis that the IGF/Akt/PTEN pathway could be modulated by the immunoproteasome. We tested this secondary hypothesis directly by stimulating this pathway in RPE cells, separate from injury, and focused solely on the question of whether immunoproteasome deficient cell signaling after IGF-1 addition differs from WT cells. As discussed above, we found that treatment of WT and immunoproteasome KO RPE cells with IGF-1 elicited a rapid activation of Akt through phosphorylation followed by a delayed temporal de-phosphorylation of Akt in the immunoproteasome KO RPE cells (Fig 7). Our ONC data demonstrated that p-Akt expression decreased after injury only in the WT-RPE cells, which could be explained by a decrease in IGF-1 levels reported previously [18]. However, data from our immunoproteasome KO mice did not follow this decrease in p-Akt after ONC indicating that either: (1) The immunoproteasome potentially regulates extracellular IGF-1 levels, or (2) the immunoproteasome regulates proteins involved in phosphorylation and/or de-phosphorylation within the cell. Our *in vivo* ONC data and cell culture data provides evidence for the immunoproteasome regulating phosphatase activity based on the differences in temporal p-Akt expression seen between WT and immunoproteasome KO animals. Protein phosphatase 2 (PP2A) is one phosphatase that has been reported to de-phosphorylate Akt [79]. It is intriguing to speculate that the immunoproteasome regulates PP2A expression or activity, and the p-Akt differences seen in both the ONC data and RPE-cell data demonstrate that animals or cells lacking the immunoproteasome have decreased PP2A activity. Further studies are needed to verify this possibility and potential signaling mechanism.

The novel results in the study have shown a significant contribution of the immunoproteasome in stress response after optic nerve injury, coinciding with previous immunoproteasome work in our laboratory and clearly establishes the immunoproteasome as a stress response protein [12,39,45,56]. This study also developed a broader understanding of signaling events that occur post-ONC in the retina, and these data provide further evidence that the immunoproteasome functions in pathways other than its canonical role of generating peptides for antigen presentation. Specifically, the PTEN/Akt pathway is regulated by immunoproteasome subunits, yet more data is needed to identify the diverging roles of each subunit. Immunoproteasome deficiency is shown be protective after ONC and adds to the growing list of potential targets for treatment in neuronal injuries. Future studies should focus on using inhibitors for the immunoproteasome subunits, such as UK-101 (LMP2) or ONC 0912 (LMP7), in dose-dependent assays to monitor the potential for axonal regeneration after injury [80]. This could provide a better therapeutic target as the immunoproteasome has been shown to modulate other stress pathways, such as NF-κB, and has a minimal constitutive role in non-immune cells prior to injury [15]. Modulating the immunoproteasome content provides a promising role in protection from disease or injury and future research should work towards elucidating the mechanisms of protection.

## Supporting Information

S1 Fig Protein Content of Shared and Standard Proteasome Subunits after Optic Nerve Crush. Protein was isolated from WT (●), L2 (■), and L7M1 (▲) mouse retinas at the indicated time points after ONC. Results are the optical density of the shared proteasome subunit α7, standard proteasome subunits β1 and β5 and are the mean ± S.E. of 3–22** mice/group compared to control (day 0). (*, p≤0.05, and #, p≤0.01 (L2) by one-way ANOVA with Dunnett’s post-test compared to the ‘day 0’ values). (** All 36 day WT mouse data represents 2 mice/group)
(TIF)

S2 Fig Ganglion Cell Counts per Total Nuclei in the GCL. Summary of the percent of ganglion cells per total nuclei (GC/nuclei) in the GCL of control mice and following ONC from WT, L2, and L7M1 mice. Results are the mean ± S.E. of 4–5 mice/group. (*, p≤0.05 by one-way ANOVA with Dunnett’s post-test compared to each cell line’s ‘control’ values).
(TIF)

S3 Fig Representative Western Blot Data from Fig 6. Protein was isolated from WT, L2, or L7M1 RPE cells after IGF-1 stimulation. Protein MW: PTEN (54kDa), pAkt (60kDa), Akt (60kDa), p-S6K1 (70 kDa), S6K1 (70 kDa). Background ‘non-specific’ bands are indicated (*).
(TIF)
