# Supplementary figures and images for: Immunoproteasome Deficiency Protects in the Retina after Optic Nerve Crush
Source: PLoS One. 2015 May 15;10(5):e0126768. doi: 10.1371/journal.pone.0126768 (PMC4433222; doi:10.1371/journal.pone.0126768)

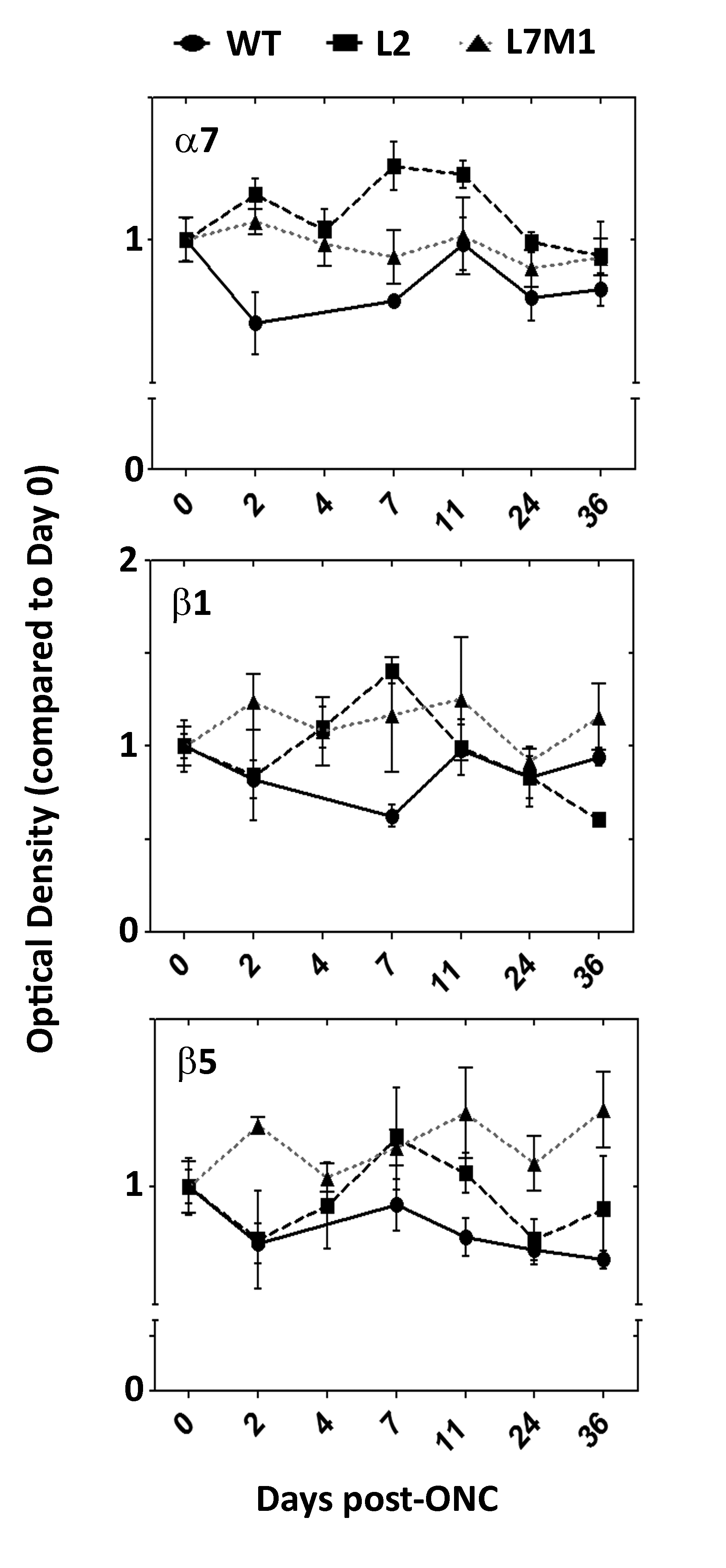

Supplement: S1 Fig — Protein was isolated from WT (●), L2 (■), and L7M1 (▲) mouse retinas at the indicated time points after ONC. Results are the optical density of the shared proteasome subunit α7, standard proteasome subunits β1 and β5 and are the mean ± S.E. of 3–22** mice/group compared to control (day 0). (*, p≤0.05, and #, p≤0.01 (L2) by one-way ANOVA with Dunnett’s post-test compared to the ‘day 0’ values). (** All 36 day WT mouse data represents 2 mice/group) (TIF) [file pone.0126768.s001.tif]

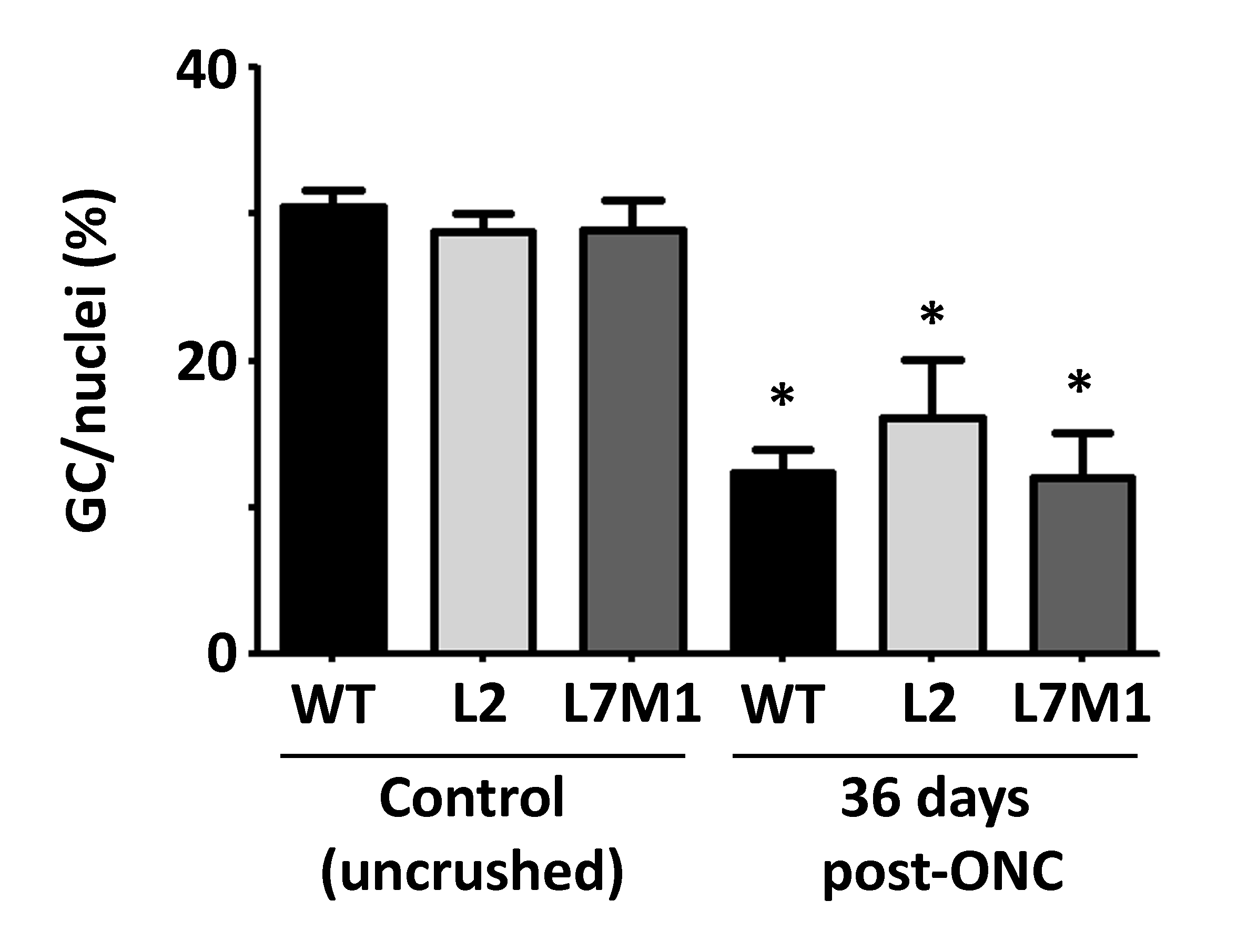

Supplement: S2 Fig — Summary of the percent of ganglion cells per total nuclei (GC/nuclei) in the GCL of control mice and following ONC from WT, L2, and L7M1 mice. Results are the mean ± S.E. of 4–5 mice/group. (*, p≤0.05 by one-way ANOVA with Dunnett’s post-test compared to each cell line’s ‘control’ values). (TIF) [file pone.0126768.s002.tif]

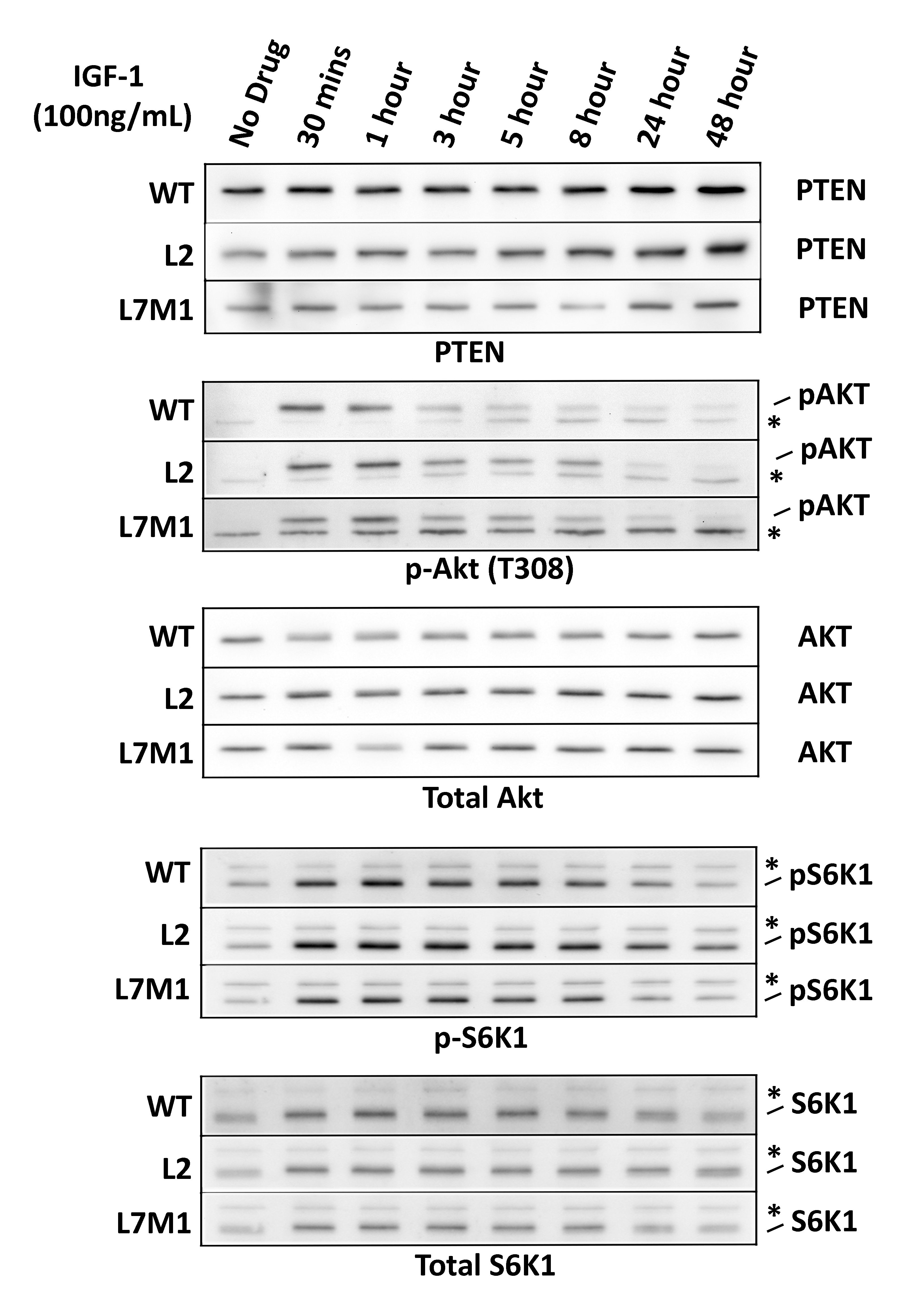

Supplement: S3 Fig — Protein was isolated from WT, L2, or L7M1 RPE cells after IGF-1 stimulation. Protein MW: PTEN (54kDa), pAkt (60kDa), Akt (60kDa), p-S6K1 (70 kDa), S6K1 (70 kDa). Background ‘non-specific’ bands are indicated (*). (TIF) [file pone.0126768.s003.tif]
